# Viruses in the Invasive Hornet *Vespa velutina*

**DOI:** 10.3390/v11111041

**Published:** 2019-11-08

**Authors:** Anne Dalmon, Philippe Gayral, Damien Decante, Christophe Klopp, Diane Bigot, Maxime Thomasson, Elisabeth A Herniou, Cédric Alaux, Yves Le Conte

**Affiliations:** 1 INRA—Unité Abeilles et Environnement, Site Agroparc, Domaine St Paul, 228, Route de l’aérodrome CS40509, 84914 Avignon Cedex 9, France; maxime.thomasson@hotmail.fr (M.T.); cedric.alaux@inra.fr (C.A.); yves.le-conte@inra.fr (Y.L.C.); 2 UMT PRADE, Unité Mixte Technologique de la PRotection de l’Abeille Dans l’Environnement, Site Agroparc, Domaine St Paul,228, Route de l’aérodrome CS40509, 84914 Avignon Cedex 9, France; d.decante.adapi@free.fr; 3 Institut de Recherche sur la Biologie de l’Insecte, UMR 7261, CNRS - Université de Tours, 37200 Tours, France; philippe.gayral@univ-tours.fr (P.G.); bigot.diane@gmail.Com (D.B.); Elisabeth.Herniou@Univ-Tours.Fr (E.A.H.); 4 ITSAP, Site Agroparc, Domaine St Paul, 228, Route de l’aérodrome CS40509, 84914 Avignon Cedex 9, France; 5 INRA—Genotoul Bioinfo, Unité MIAT Mathématiques et Informatique Appliquées, 24 chemin de Borde-Rouge, 31326 Auzeville, France; christophe.klopp@inra.fr

**Keywords:** *Vespidae*, invasive species, honey bee viruses, new viruses, DWV, ABPV, BQCV, KBV, ALPV, BeeMLV

## Abstract

The Asian yellow-legged hornet *Vespa velutina nigrithorax*, a major predator of honeybees, is spreading in Europe in part due to a lack of efficient control methods. In this study, as a first step to identify biological control agents, we characterized viral RNA sequences present in asymptomatic or symptomatic hornets. Among 19 detected viruses, the honey bee virus *Deformed wing virus-B* was predominant in all the samples, particularly in muscles from the symptomatic hornet, suggesting a putative cause of the deformed wing symptom. Interestingly, two new viruses closely related to *Acyrthosiphon pisum* *virus* and *Himetobi P* *virus* and viruses typically associated with honey bees, *Acute bee paralysis virus* and *Black queen cell virus*, were detected in the brain and muscles, and may correspond to the circulation and possible replication forms of these viruses in the hornet. *Aphid lethal paralysis virus*, *Bee Macula-like virus*, and Moku virus, which are known to infect honey bees, were also identified in the gut virus metagenome of hornets. Therefore, our study underlined the urgent need to study the host range of these newly discovered viruses in hornets to determine whether they represent a new threat for honey bees or a hope for the biocontrol of *V. velutina*.

## 1. Introduction

The original distribution area of the Asian yellow-legged hornet *Vespa velutina nigrithorax*, native to Southeast Asia, ranges from Nepal to South Eastern China. However, in 2004, it was first observed near Agen, France, [1], and one year later, phylogenetically identified [2,3]. Its accidental introduction, through very few or single multi-mated gynes, most probably occurred before 2005 via boat transport of bonsai pots from the Zhejiang or Jiangsu provinces of Eastern China [4,5].

Since a single individual can found a colony, this outbreak had a high invasive potential, even though, like other social *Vespidae* under temperate climates, the colonies last only one season. Every year, the surviving yellow-legged hornet-mated queens disperse in order to initiate new nests after hibernation. Each colony, initiated by this single individual, develops until the following autumn, first producing several thousand workers, and then hundreds of males and new founders, which mate in autumn [6]. Despite the genetic bottleneck and inbreeding depression associated with this single introduction event [4,5,7], no population extinction has been observed yet. Instead, this first successful introduction of an exotic *Vespidae* in Europe resulted in a rapid colonization of France and neighbouring countries (Spain, Portugal, Italy, Belgium, Germany, England, and the Netherlands; INPN, 2019), with substantial population levels and impacts in most of the colonized regions [5,8,9,10]. Moreover, the niche models developed in order to determine its possible distribution area [1,11,12,13,14,15,16,17] converge to predict a much wider distribution in the future.

During nest development, *V. velutina* predates on a wide diversity of insects such as hoverflies and houseflies, but with a preference for aggregative species, including social Hymenoptera species such as honey bees and common wasps [18]. Although the mechanisms resulting in colony collapse remain poorly described, predictive models suggest that high *V. velutina* pressure strongly impacts the colonies [19]. To date, empirical methods, including spring foundress trapping and autumnal worker mass trapping, have been used to impair *V. velutina* establishment and dissemination [20,21]. However, the efficacy of these methods is insufficient to control *V. velutina* populations [18,22,23,24,25,26]. Nest destruction seems to be efficient, but despite recent technological advances for nest detection [27,28,29], these are cumbersome and expensive [30], thus limiting large-scale implementation. 

As observed with other wasp introduction events [31], the lack of efficient control methods stems from a lack of knowledge on the biology, behaviour and pathology of *V. velutina* [6,9,21,23]. Regarding pathogens that could affect the Asian hornet, only *Deformed wing virus* [32], Moku virus [33] and *Israeli acute paralysis virus* (IAPV) [34] have been described in some Asian specimens. However, 11 viruses initially detected in managed Western honey bees (*Apis mellifera*) have also been identified in wild Hymenoptera species [35] including *Vespula* spp. [36,37,38], which belongs to the same sub-family as the *Vespa* genus, *Vespinae*. Because *V. velutina* is an important predator of *A. mellifera*, we can expect pathogen transfers from target prey to predator. Among the seven viruses commonly found in honey bee colonies, *Deformed wing virus* (DWV), *Black queen cell virus* (BQCV) and *Sacbrood virus* (SBV) have the highest prevalence, as they are detected in up to 90% of western honey bee colonies [39,40,41]. DWV presence has been positively correlated with colony losses [41,42,43,44,45]. Different genotypic variants have been described: the original first described DWV named DWV-A [46], *Varroa destructor virus 1* (VDV-1) recently renamed DWV-B, and DWV-C [47]. Acute paralysis viruses from the “AKI” complex (*Acute bee paralysis virus* (ABPV), *Kashmir bee virus* (KBV), *Israeli acute bee paralysis* (IAPV)) have a worldwide distribution. While ABPV is more prevalent in Europe and South America, KBV prevails in North America and South America and IAPV in the Middle East and Australia [48]. *Chronic bee paralysis virus* (CBPV) is widespread, although not highly prevalent in western honey bees [49], but has been detected in other social insects such as ants [50]. Such widespread viruses initially discovered in bees may therefore be transmitted to the predator *Vespa velutina*, which may also be affected by some specific pathogens.

In this study, we investigated viral agents of *V. velutina* in order to identify specific viruses or strains that could be used as potential biocontrol agents of *V. velutina* populations. A number of viral microbial control agents have been successfully used against invasive species [51] or pathogens [52]. Therefore, we searched for any viral sequences using RNA sequencing in order to detect either undescribed viruses or viruses already described in other host species. To further investigate virus infection and whether some are tissue-specific, RNA-seq analysis was performed on different body parts. Lastly, we analysed the genetic diversity of the honey bee viruses from both hornet and bee samples to infer possible host-specific strains.

## 2. Materials and Methods 

### 2.1. Asian Hornet and Honey Bee Sampling

**Figure 1:**
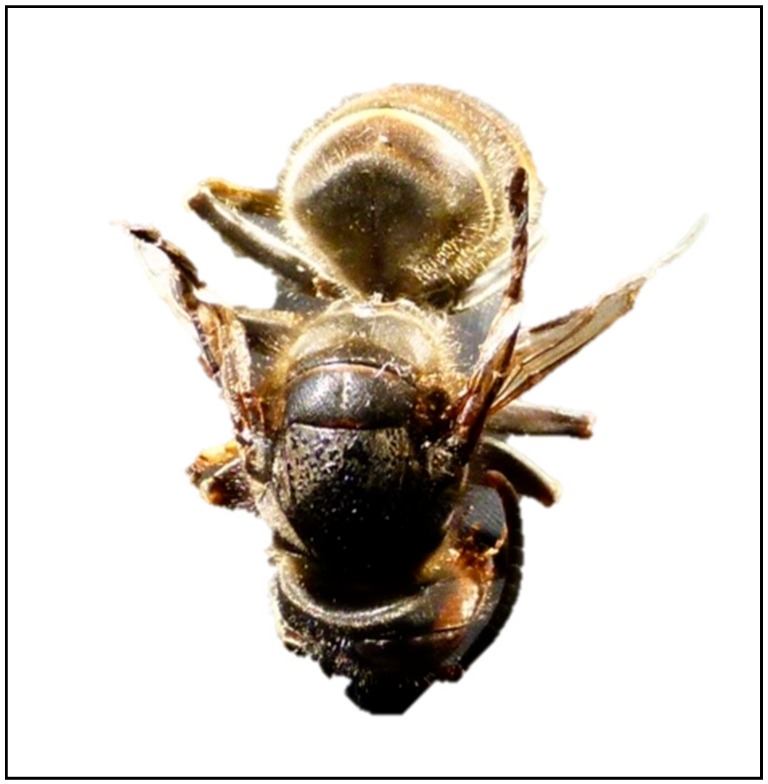
Dorsal view of deformed wings in hornet (sample 159+ naturally curled up by freezing).

Hornets were sampled on five occasions (one to four hornets per sampling) in front of honey bee colonies in two apiaries from the south-east of France during autumn 2014 (Table S1). Live hornets were kept at room temperature for 3 to 4 days and provided with water and sugar syrup *ad libitum* to foster elimination of previous feeding before dissections and then frozen at 20 °C. One hornet sampled by a beekeeper (in southeast France) exhibited deformed wings (159+, Figure 1) and was similarly frozen at −20 °C. Pools of bees from the apiaries where hornets were preying on were simultaneously collected (Table S1) and immediately frozen at −20°C in the laboratory. All samples were then stored at −80 °C.

Dissections of two asymptomatic hornets (individuals 140-1 and 140-2) and the hornet 159+ exhibiting deformed wings were performed on ice and resulted in brain, muscle, gut and mandible samples. The remaining external parts (cuticle and legs) were pooled together. For the deformed wings hornet, only muscle, intestine and external parts could be sampled (Table S1). For the dissection of other body parts, we used RNAlater^®^-ICE (Ambion, ref # AM7030). Head, thorax and abdomen of non-symptomatic individuals from the same apiary (140-3 and 144-1) were cut (avoiding thawing) and separately incubated in 1.7 mL RNAlater^®^-ICE at −80 °C for 18 h, then for 20 h at −20 °C. We then dissected the gut, the abdomen cuticle and fat bodies, and the six legs, which where pooled with the wings and thorax cuticle, the thorax muscles, the mandibles and the brain. Leftover samples of thorax and head were also kept for later analysis. The detailed list of the hornet and honey bee samples can be found in Table S1.

### 2.2. RNA Extraction

All the hornet samples (whole, or dissected tissues) were ground in 500 to 800 µL TRIzol^®^ Reagent (Invitrogen, ref #15596-026, Carlsbad, California, USA), depending on sample size) with a 0.8 cm-diameter. bead in a 2 ml tube and a TissueLyser (Qiagen) (four times for 30 s each at 30 Hz and 30 s intervals). The tube was then centrifuged for 2 min at 12,000× *g* at 4 °C, and the supernatant was collected into a new tube to be processed for RNA extraction. RNA was extracted using an RNeasy mini kit (Qiagen, Courtaboeuf, France) and DNase according to the manufacturer’s instructions. The final suspension volume was 100 µL for whole hornet and 50 µL for the dissected parts. RNA was quantified with a Nanodrop2000^®^ (Thermo Scientific, Waltham, Massachusetts, USA) and RNA quality was assessed with RNA Chips (Agilent technologies, Santa Clara, California, USA) using an Agilent 2100 Bioanalyzer.

### 2.3. Next Generation Sequencing (NGS)

Next generation RNA-sequencing was performed by MGX-Montpellier GenomiX (Montpellier, France) on four samples showing the best RNA yield and quality: muscle from the symptomatic specimen (159M), and brain (140C), muscle (140M) and gut (144I) from non-symptomatic specimens. To enrich the samples for polyA and mRNA, ribosomal RNA was first depleted twice with a Ribo-zero kit (Magnetic Gold Epidemiology, Illumina Netherlands, Eindhoven, The Netherlands). The library was prepared with a TruSeq Stranded mRNA Sample Preparation kit (Illumina). Samples were paired-end sequenced (2 × 125 nt) with a Illumina HiSeq 2500 sequencer. 

### 2.4. General Analysis of Viral Content

Short reads were quality checked (fastQC and contaminant search including *E. coli*, yeast and phiX) and stored in ng6.toulouse.inra.fr [53]. Reads were adapter-cleaned using cutadapt [54] and each of the five libraries was independently assembled with Oases [55] (version 0.2.06 using 25, 31, 37, 43, 49, 55, 61, 65, 69 kmers). The assembled contigs were merged and the overlapping ones removed using CD-HIT-EST [56] (Version v4.6.1-2012-08-27 with parameter -c 1). The Sequence Read Archive in Genbank is accessible with SRA accession: PRJNA556788.

For a first general analysis of viral content, all contigs were renamed using their sample name as the prefix and gathered in a unique file. This file was reduced by CD-HIT-EST in the same way. The resulting contigs were annotated with kraken [57] (version 0.10.5-beta with standard parameters). Kraken viral annotations were manually checked and only contigs with at least 20 kmers-long specific blocks for the set of viruses presented in Figure S1 were kept for further analyses. The number of contigs and their identifier are presented in Table 1. The reads were remapped to the global contig set using bwa mem [58] (version 0.7.12-r1039 with standard parameters), sorted and compressed using samtools [59] (view, sort and index version 1.1 with standard parameters) and the coverage of the selected contigs was visually inspected in IGV. 

### 2.5. Analysis of Viral Genomes 

A two-step mapping method was used as in previous virus discovery studies in mosquitoes [60] and wild bees [61] as this improves the recovery of full-length genomes and ensures that final contig assembly stems from reads originating from the hornet viral metagenome only. In short, in order to reconstruct virus genomes from the four assembled RNA sequencing datasets, full and partial ORFs were first detected in hornet contigs using Prodigal V2_60 software for metagenomic data [62]. Contigs with relevant virus homologies (e-value < 10^−5^) were then detected using BLASTx program [63] on the non-redundant (nr) protein database from the NCBI (National Center for Biointechnology Information). For each of the four libraries, assembled contigs matching expected virus genome size, or the presence of multiple viral hits belonging to the same viral family were an indication of putative viral genomes to be reconstructed.

Virus genome reconstruction was performed in three steps. First, for each potential virus found, the nucleotide genomic sequence of the virus species detected by BLAST as the first hit was retrieved from GenBank and used as a viral reference genome for further reconstruction. Paired-end reads from each library were mapped using Bowtie2 V2.3.3 [64] against all viral reference genomes, and only the best matches were reported. When necessary, the consensus sequences of the mapped reads were re-assembled with the initial contigs to fill the gaps. Secondly, the reads were mapped again on the consensus sequences of the targeted virus obtained at the first step. Thirdly, ORFs were predicted using the ORF finder program of the Geneious 9.1.8 software [65] and annotated using InterProScan version 5, [66], CD-search web using the CCD 3030 database and e-value < 0.05, and BLASTp (e-value < 0.001) against the nucleotide database. The sequences have been submitted to GenBank (accession numbers MN565031 to MN565059).

A more stringent mapping was also performed to calculate the mean coverage and expression level FPKM of the reconstructed genome and to estimate the quantity of polymorphism. For this mapping, paired-reads ends were trimmed (five bases at 5′ ends and 9 bases at 3′ ends) using the fastx-trimmer program from the standalone FASTX-Toolkit 0.0.13 when the median quality showed the lowest values (Q < 38) and when the quality variance was high. The number of bases trimmed was chosen according to Illumina Quality plots generated with fastx_quality_stats and Fastq_quality_boxplot_graph.sh programs implemented in the FASTX-Toolkit (Figures S2 and S3). Trimmed paired-end reads were mapped against the reconstructed genomes using Bowtie2 as described above. Polymorphism *Pi* statistics was estimated using a Popoolation software with the disable correction parameter set to 1 [67]. To analyse the specific single nucleotide polymorphism (SNP) distribution of the reconstructed virus genomes across the samples, SNP detection was performed using Geneious Prime 2019 software, using a minimum coverage of 10X, the minimum variant frequency set at 0.3 and the other parameters set by default.

A Spearman correlation test between polymorphism and expression level was performed using the PAST v3 program [68], using 0.05 as the significance threshold.

### 2.6. Phylogenetic Analysis

Sequences were aligned with the Muscle program [69]. Site selection was performed using GBlocks [70] available in Seaview 4.4.4 suite [71] when necessary. Maximum likelihood phylogenies were built using the PhyML 3.0 program [72], except for DWV complete genomes, for which we used SplitsTree4 [73] to represent putative recombination events. The best substitution models for nucleotide and protein substitution were first assessed using the SMS [74] program and AIC selection criterion. Branch supports were calculated using a LRT SH-like likelihood-based method. Depending on the amount of published sequence data of closely-related sequences, trees were built either at the species level from nucleotide whole genome sequences alignments, or at a broader taxonomic level (typically genera or families) from RNA-dependent RNA polymerase (RdRp) ORF or domain amino acid alignments.

### 2.7. RT-PCR

Reverse transcription of either 1 µg RNA from the whole hornet samples or 144 ng to 1 µg RNA from the dissected parts was performed in 20 reaction volume using random priming and according to the manufacturer’s protocol (High capacity RNA to cDNA, Life technologies).

Multiplex PCR was performed as previously described [75,76] to detect DWV, SBV, BQCV, CBPV, ABPV, KBV and IAPV. Briefly, an 8-µM master mix of the 11 primers was prepared and stored at −20 °C. Half of the cDNA (10 µL) was added to a mix of 0.5 µM primers, 2.5 mM MgCl2, 0.2 mM dNTPs and 2.5 U of Go Taq^®^ G2 Flexi DNA polymerase (Promega) in 25 µl total volume. PCR reactions were performed using an Eppendorf thermocycler. The following program was used: one cycle at 94 °C for 5 min, 35 cycles at 94 °C for 30 s, 56 °C for 30 s, and 72 °C for 45 s, and a final elongation cycle at 72 °C for 10 min. The reactions were then held at 10 °C. Five microliters of PCR products were run in 4% agarose gel, or one microliter was analysed with DNA Chips using an Agilent DNA 1000 reagent (Agilent technologies). The presence of the virus was determined according to the expected sizes of the fragments (IAPV: 158 bp; DWV: 269 bp; SBV: 342 bp; ABPV: 460 bp; BQCV: 536 bp; KBV: 641 bp; CBPV: 774 bp). 

For SANGER sequencing, PCR was carried out with specific virus primers in the same conditions, except for DWV sequencing, which was performed from the (conserved) helicase coding region for partial sequencing as described in [77]. PCR products quality (purity and band size) was controlled in 1% agarose gel prior to sequencing. The PCR products were sent to GenoScreen (France) for purification and Sanger sequencing.

## 3. Results

### 3.1. Virus Discovery from RNAseq Experiments

RNAseq data were used for identifying viruses from hornet tissues sampled from one symptomatic and two asymptomatic hornets (brain, muscle or gut, Table S1). Initially, 1.7 M contigs were assembled, and after redundancy removal, 509,572 contigs could be annotated with Kraken: 113,336 sequences were classified (22.24%), and 396,236 sequences resulted unclassified (77.76%) (Table 1).

Overall, 19 viral species, including seven complete genomes and 12 partial sequences, could be identified from the four libraries (Table 2). Apart from honey bee-associated viruses, a high number of contigs was initially assigned as dsDNA viruses in brain, muscle, or intestine samples (Figure S1) in spite of DNase treatment during RNA extraction. However, further BLAST analyses suggested that these sequences corresponded to insect genes comprising conserved functional domains shared between eukaryotes and large dsDNA viruses. Indeed, no assembled contigs from this category allowed the reconstruction of full-length or even partial virus genomes.

Of the 509,572 virus contigs identified by Kraken, DWV-B (=VDV-1) was clearly predominant both in muscle sample 159M (40.3% of the classified contigs) and gut sample 144I (93.2% of the classified contigs) (Figure S1).

### 3.2. DWV-B is Highly Prevalent in Symptomatic and Asymptomatic Hornets

Firstly, complete DWV-B genomes could be reconstructed from the RNA libraries of individual organs (muscles 159M, gut 144I, brain 140C) (Table 2, Figure S4a–d). In the symptomatic sample 159M, the DWV-B virus was the only virus detected and no other viral contig could be found as compared to the other libraries.

**Figure 2:**
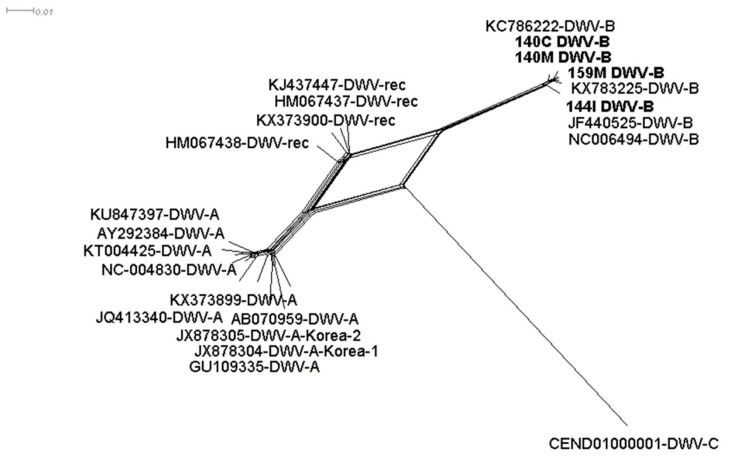
Split tree of *Deformed wing virus* from 23 nucleotide sequences. All ambiguous positions were removed for each sequence pair (pairwise deletion option). There was a total of 7560 positions from the 3’ end of the DWV genome in the final dataset. GenBank accession numbers and samples 140C (brain), 140M and 159M (muscle) and 144I (intestine) are associated with the DWV strain. Contigs from the intestine sample assigned as DWV-C were too short to be included.

Phylogenetic analysis from the DWV-B assembled contigs showed that the brain (140C), muscle (140M, 159M), and gut (144I) samples clustered together with the DWV-B reference sequence (NC006494-DWV-B) and not with DWV recombinant variants (HM067437, HM067437, KJ437447 and KX373900) (Figure 2). In the library, 144I various contigs were found to correspond to the DWV-C strain but in this case, the genome was segmented (Table 2).

Secondly, multiplex PCR was used to study the presence of DWV in both hornets and honey bees sampled at the same location. DWV was detected from all the hornets and bee samples except for the dissected parts of sample 140-1, where only gut and muscle were positive (Table 2, Figure S5).

In order to determine whether the DWV strains in hornets and bees sampled together were the same, 11 helicase coding regions were sequenced. This confirmed that all the sequences from this study belonged to DWV species (Genbank Submission 2248150), but no strongly supported clusters could be identified in the phylogenetic tree because helicase is too conserved for fine phylogenetic resolution.

### 3.3. Six Other Bee-Associated Viruses Detected in Hornets by RNAseq

**Figure 3:**
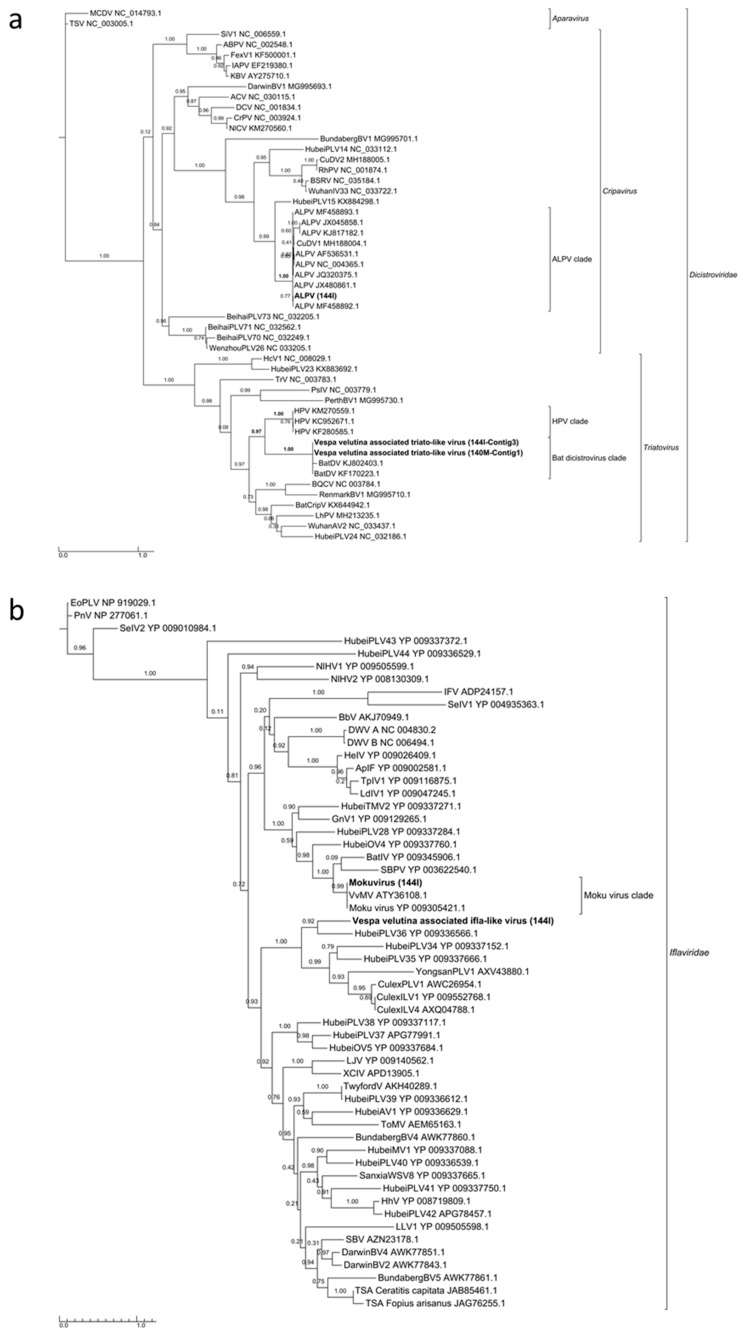
Maximum likelihood phylogeny of the replicase genes of (**a**) ALPV and *Vespa velutina* associated triato-like virus (372 aa, LG+I+G+F model), and (**b**) Moku virus and *Vespa velutina* associated ifla-like virus (338 aa, LG+I+G+F model) detected in hornets. The complete virus names are shown in Table S2.

*Aphid lethal paralysis virus* (ALPV, *Dicistroviridae*, *Cripavirus*), Moku virus (*Iflaviridae*, *Iflavirus*) and a *Bee macula-like virus* (BeeMLV, unclassified *Tymoviridae*) were found in the hornet gut (144I). Complete genome could not be assembled for these viruses, but 4.2, 6.3 and 3.1 Kb contigs of the corresponding genomes could be assembled (Figure S6a–c). Phylogenies from the RdRp domain clearly showed that these viruses belonged to the known ALPV, Moku virus and BeeMLV species (Figure 3a,b and Figure S7).

*Kashmir bee virus* (KBV, *Dicistroviridae*, *Aparavirus*) was found in the brain (sample 140C), but not in the muscle of the same hornet individual. A partial 3.5-kb genome could be assembled (Figure S6d), but the RdRp domain was too short to produce a qualitative alignment and no phylogenies could be built. However, homology search strongly suggested that this sequence belonged to KBV species (best BLASTx hit with, for example, one High-Scoring Segment Pair displaying e = E–100, 94% identity over 560 amino acids).

A 9.5-Kb complete genome of *Acute bee paralysis virus* (ABPV, *Dicistroviridae*, *Aparavirus*) and an 8.5-Kb complete genome of Black queen cell virus (BQCV, *Dicistroviridae*, *Triatovirus*) were found in the gut of hornet (144I) (Figure S6e–f). The taxonomic assignment of both ABPV and BQCV was again clearly confirmed by RdRp phylogeny (Figure S8).

### 3.4. Eleven Other Insect Viruses Detected in Hornets from RNAseq

**Figure 4:**
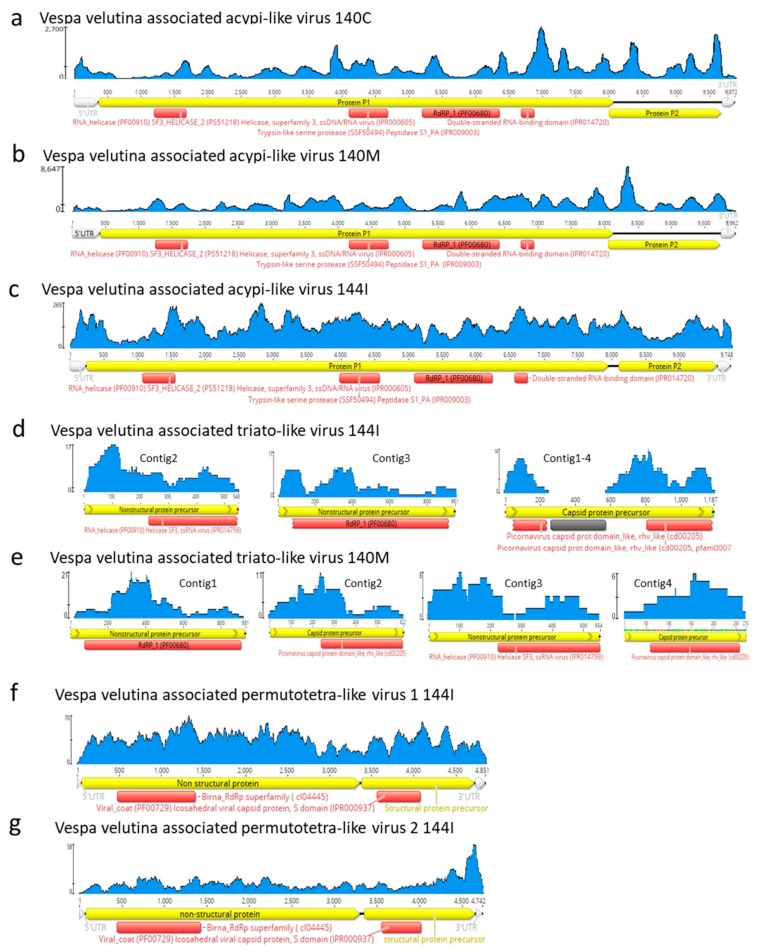
Schematic representation, coverage and annotation of the complete or partial genomes of insect RNA viruses found in hornets. (**a**–**c**) *Vespa velutina* associated acypi-like virus; (**d**–**e**) *Vespa velutina* associated triato-like virus and (**f**–**g**) *Vespa velutina* associated permutotetra-like virus 1 and 2.

**Figure 5:**
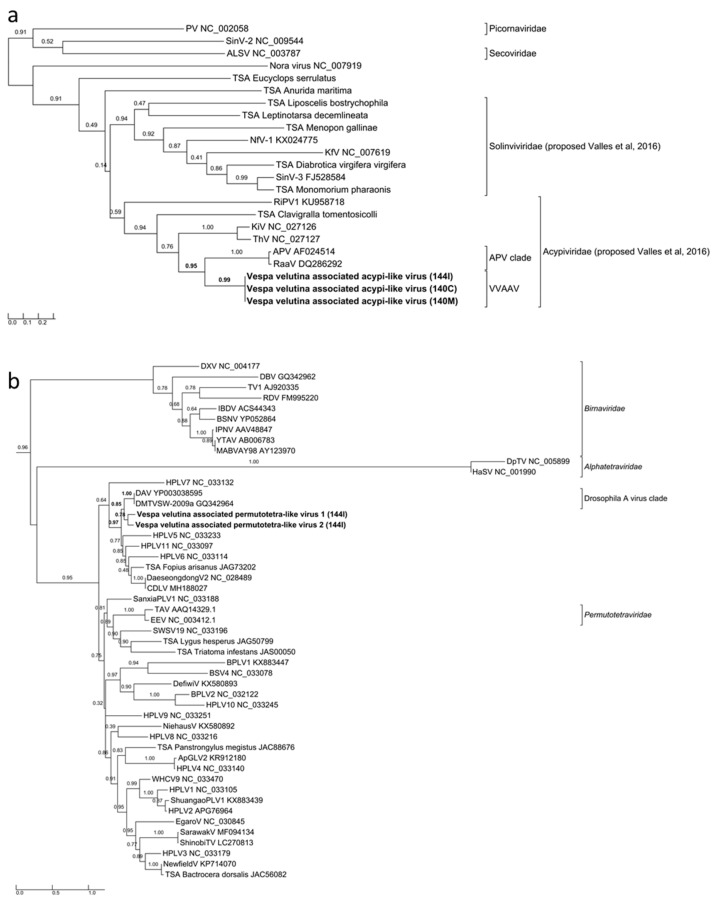
Maximum likelihood amino acid replicase phylogeny of (**a**) *Vespa velutina* associated acypi-like virus (277 aa, RtREV +G+I+Fmodel), and (**b**) *Vespa velutina* associated permutotetra-like virus 1 and 2 (219 aa, LG+G model). GenBank accessions are shown in Table S2.

Three complete 9.7–9.9 Kb genomes of *Vespa velutina* associated acypi-like virus were detected in both the brain (140C) and muscle (140M) from the same hornet and in the gut of the 144I individual (Table 2, Figure 4a–c). The phylogenetic tree indicated that all three isolated *Vespa velutina* associated acypi-like virus clustered in a single clade (aLRT node statistics = 0.99) and showed similarities to the *Acyrthosiphon pisum* virus and the Rosy apple aphid virus, both proposed as Acypiviridae (node aLRT statictic = 0.95) (Figure 5a). The *Vespa velutina* associated acypi-like virus genome contained two ORFs. ORF1 product contained sequence motifs characteristic of Helicase (superfamily 3, PR000605) and RNA-dependent RNA polymerase (PF00680), a genomic organization similar to that of APV [78] (Figure 4a–c). Thus, *Vespa velutina* associated acypi-like virus is most certainly a new hornet virus species from the *Picornavirales* order and belongs to the newly proposed Acypiviridae family.

*Vespa velutina* associated triato-like virus was identified from hornet muscle (140M) and gut (144I) (Table 2, Figure 4d–e). Coverage for *Himetobi P virus* (HPV)-like sequences did not allow the reconstruction of the whole virus genome, but only four annotated contigs from both muscle (140M) and gut (144I) samples, covering 2.4 kb. RNA-dependent RNA polymerase domains (PF00680) were identified in contig 1 and 3 from samples 140M and 144I, respectively, and phylogeny clearly showed that both *Vespa velutina* associated triato-like viruses belonged to genus *Triatovirus*, and are closely related to the *Bat dicistrovirus* species (node aLRT = 1) (Figure 3a). Along with *Bat dicistrovirus*, *Vespa velutina* associated triato-like virus is a sister group of the bee virus *Himetobi P virus* (HPV, *Dicistroviridae*, *Triatovirus*) (node aLRT = 0.97) and shares only 50% identity with HPV.

The remaining nine insect viruses were only detected in the gut transcriptome of 144I, and not in brain or muscle tissues. These viruses likely originate from infected preys ingested by hornets, and not from infected hornets themselves. *Vespa velutina* associated permutotetra-like virus 1 and 2 are two full-length genomes of 4.7–4.8 Kb (Table 2). The genomic organization of *Vespa velutina* associated permutotetra-like virus 1 and 2 was similar to that of the *Drosophila* A virus (unassigned) and the other permutotetra-like viruses [79] (Figure 4f–g). RdRP phylogeny showed that *Vespa velutina* associated permutotetra-like virus 1 and 2 are two distinct species and are the sister group of the *Drosophila* A virus (Figure 5b).

**Figure 6:**
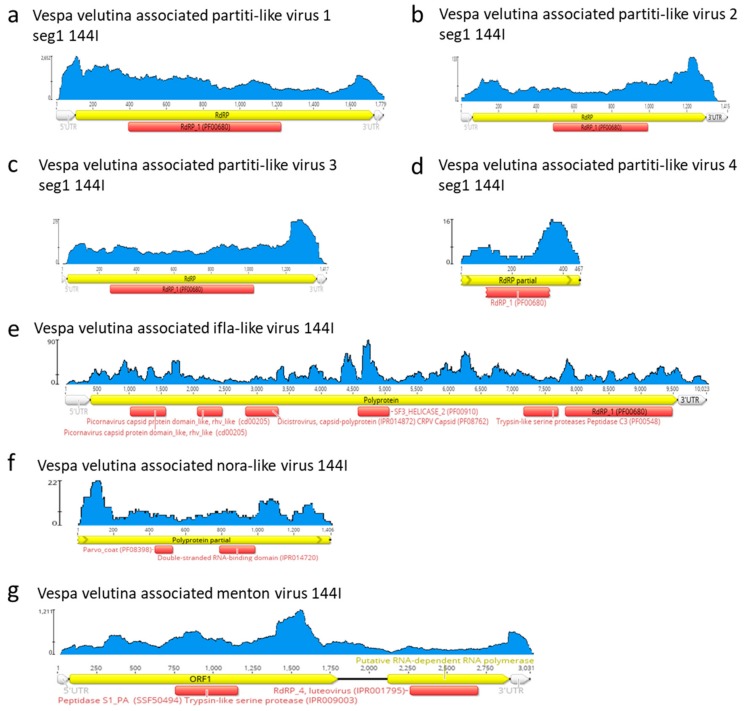
Schematic representation, coverage and annotation of complete or partial genomes of insect RNA viruses found in hornets. (**a**–**d**): *Vespa velutina* associated partiti-like virus 1-4, (**e**): *Vespa velutina* associated ifla-like virus, (**f**): *Vespa velutina* associated nora-like virus, (**g**): *Vespa velutina* associated Menton virus.

**Figure 7:**
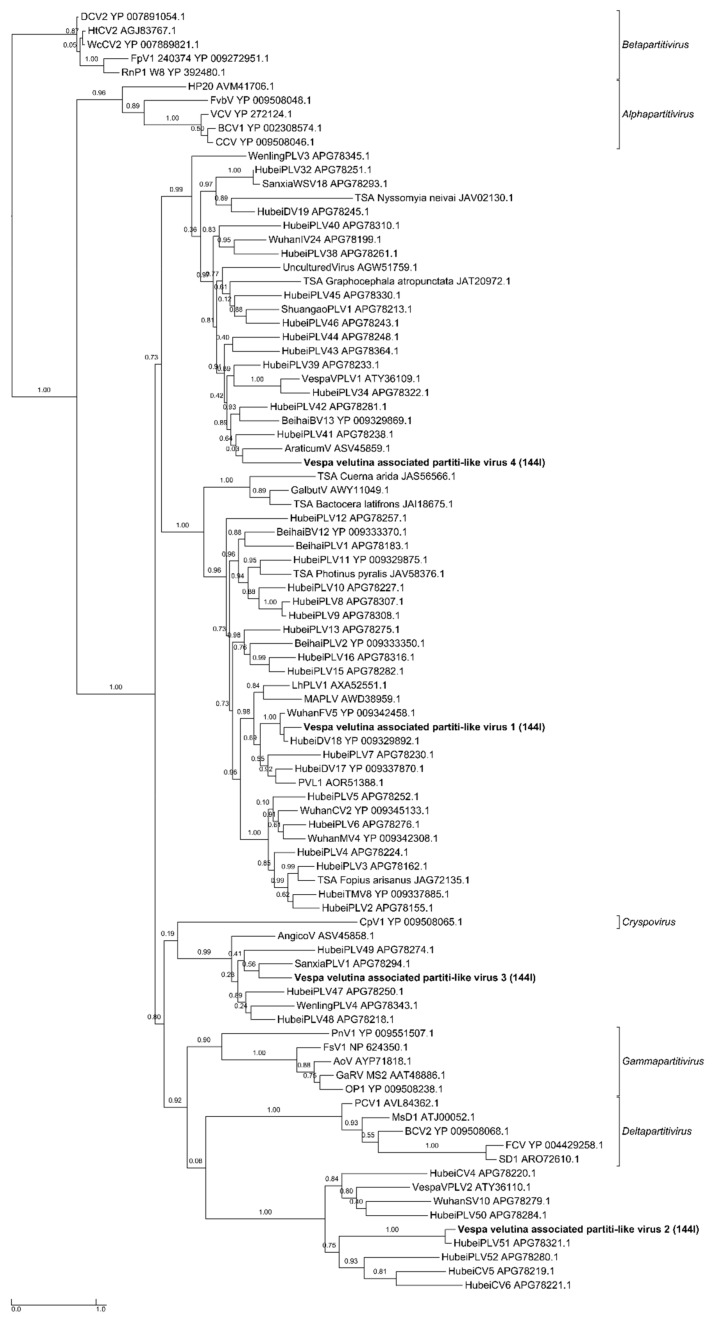
Maximum likelihood amino acid replicase phylogeny of *Vespa velutina* associated partiti-like viruses 1–4 (384 aa, LG+I+G+F model). GenBank accessions are in Table S2.

*Vespa velutina* associated partiti-like viruses 1–3 are three full-length genomic segment 1 of 1.4–1.8 Kb, (Table 2, Figure 6a–d). *Vespa velutina* associated partiti-like virus 4 is fragmented, but its 0.5 Kb contig contained RdRp domain, which enabled us to build a phylogeny. Figure 7 shows that these four assembled genomes belonged to four new viruses of distinct genera and are distributed across the *Partitiviridae* tree.

**Figure 8:**
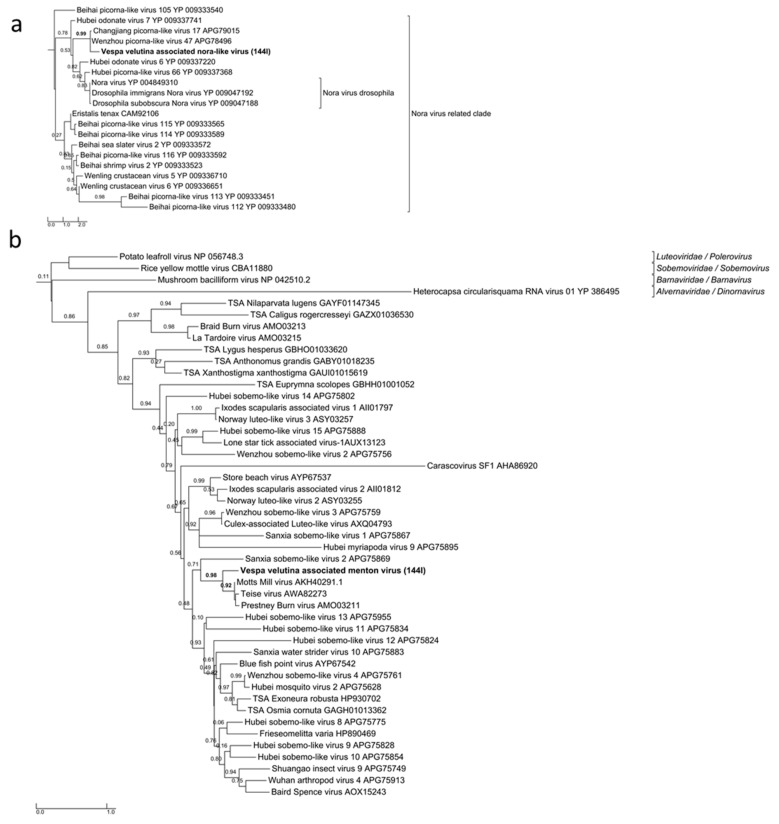
Maximum likelihood amino acid replicase phylogeny of (**a**) *Vespa velutina* associated nora-like virus (54 aa, LG+G model), (**b**) *Vespa velutina* associated Menton virus (162 aa, LG+G model). GenBank accessions are shown in Table S2.

*Vespa velutina* associated ifla-like virus is a 10-Kb full-length genome (Table 2, Figure 6e). Replicase phylogeny indicated that this new virus is a member of the *Iflaviridae* family (Order *Picornavirales*) (Figure 3b). *Vespa velutina* associated nora-like virus is a 1.4-Kb genomic fragment containing parvo coat and dsRNA-binding domains (Table 2, Figure 6f). Maximum likelihood phylogeny showed that this virus is closely related to the Nora virus discovered in the *Drosophila* species [80], a member of a newly described picorna-like virus family infecting arthropods [81] (Figure 8a).

*Vespa velutina* associated Menton virus is a 3-Kb full-length genomic segment 1 containing serine protease and RdRp domains (Table 2, Figure 6g). Phylogeny indicated that this virus was a close relative of Motts Mill virus, Teise virus and Prestney Burn virus, detected in several *Drosophila* species [82,83,84] (Figure 8b). These viruses are members of an yet unclassified new family related to *Luteoviridae*, *Sobemoviridae*, *Barnaviridae* and *Alvernaviridae*.

### 3.5. Viral Polymorphism Study Confirmed Genuine Hornet Infections

Viral nucleotide diversity was estimated using Pi polymorphism in the three virus species found in two or more samples, i.e., *Vespa velutina* associated acypi-like virus, DWV-B, and *Vespa velutina* associated triato-like virus. For a given virus species, polymorphism levels were clearly distinct between samples, and a two-fold change was observed between the brain and intestine samples in *Vespa velutina* associated acypi-like virus (Table S3). Furthermore, Pi estimates were on average higher in intestine samples compared to the other organs. Finally, we confirmed that Pi estimates were not biased and influenced by a difference in viral genomes coverage and showed that Pi was not correlated with Fragment per Kilobase of viral sequences per million reads (FPKM) expression levels (Spearman’s rho = 0.1, *p* = 0.78). These results show that viral sequences found in brain and muscles have different genetic diversity signatures compared to the ones found in intestine, and thus that they did not result from simple cross-contaminations.

To further investigate this point, specific patterns of single nucleotide polymorphisms (SNPs) were analyzed in *Vespa velutina* associated acypi-like virus. DWV-B and *Vespa velutina* associated triato-like virus were not included in this analysis since conditions on coverage and/or minimum variant frequency thresholds were not met. Table S4 shows the eight loci distributed across the viral genome were the SNP patterns highlighted in bold were clearly different between samples. In six loci, both brain and muscle samples shared the same SNP patterns, whereas intestine displayed distinct SNPs. Moreover, the SNP patterns were different in the two other loci (SNPs numbers 6.089 and 8.597) between the brain and muscle from the same hornet individual.

### 3.6. Distribution of Bee-Associated Viruses in Hornets Detected by Multiplex RT-PCR

Eight hornet samples were positive for SBV, as determined by RT-PCR (Table S1). A similar pattern of SBV presence was found in three honey bee pools sampled at the same locations than SBV-positive hornets (bee-pool-142, -145, -149). Only the gut, and no other dissected parts, was positive for SBV in 140-2 hornet (Table S1).

From the multiplex PCR method, eleven hornets out of twelve tested positive for ABPV, (Table S1). However, ABPV was detected by PCR in all the dissected parts of hornet 140-2 and both in the muscle and gut of hornet 140-1. Partial sequences from dissected parts were completely similar, even though they were sampled from two hornets and showed 99% identity with *Acute bee paralysis virus* isolate Hungary 1 (GenBank AF486072.2) isolated from bees (Genbank Submission 2248150). However, they were different from other ABPV sequences obtained from bees in the same experiment (29 informative positions out of 431). 

Seven hornet samples were positive for BQCV, as determined by RT-PCR, and this virus was detected not only in the gut, as suggested from RNA-sequencing data in the sample 144I, but also in muscles (hornet 140-1) (Figure S6e, Table S1). 

For both BQCV and ABPV, similar patterns of BQCV or ABPV presence or absence were found in bee samples at the same locations than hornets (Table S1). 

## 4. Discussion

Polymorphism and SNP analyses of viral signatures between samples indicated that the detection of the *Vespa velutina* associated acypi-like virus, DWV-B, and *Vespa velutina* associated triato-like virus sequences in the brain and muscle samples were not a result of simple cross-contamination that could have occurred during sample processing or library preparation. Instead, this study indicates that viruses typically associated with honey bees were actively infecting several hornet tissues. To date, oral inoculation has been the only known virus transmission route in hornets. The presence of viruses in the brain and muscle tissues combined with intra-species levels of polymorphism (SNP variation) indicate virus multiplication in hornets. The putative DWV circulation in the insect was further emphasized by the identification of DWV in other organs like mandibles or thorax cuticle and legs. Furthermore, DWV-B was shown to be predominant in all the RNA-sequencing samples. DWV-B read counts in the muscle sample were much higher in the hornet exhibiting symptoms of deformed wings (159M) than in the sample that did not show any symptoms (140M). The high DWV-B expression level may be related to the symptoms, when a low level of expression of the virus may correspond to a latent infection, as defined by de Miranda and Genersch [85], especially since DWV-B has been characterized, in vitro, as a more virulent honey bee virus strain than DWV-A [86]. However, one needs to be cautious regarding this assumption since only one hornet with a deformed wing symptom was analyzed. DWV-C was detected in a low number and only in hornet gut, which suggests food contamination or latent infection. Furthermore, since only partial sequences of DWV-C could be assembled, we could assume that they resulted from recombination with other DWV strains [77]. To our knowledge, this is the first description of DWV-C in France since its description in UK [46]. Finally, when comparing DWV-B sequences from hornets to previously described DWV sequences from honeybees, they seem to be very close, which raises the question about the historical host range of DWV. The detection of DWV combined with genotypic diversity studies in the native area of *V. velutina* would clarify whether *V. velutina* is an usual host for DWV or if it only became infected from *A. mellifera* honeybees after its introduction in Europe.

ABPV and, to a lesser extent, BQCV were both identified in muscles and/or cuticle, legs, and mandibles, as well as in the guts. Similarly, food contamination alone seems unlikely for the presence of these viruses in hornets. BQCV is known to infect many *Apis* species (up to 90% prevalence in *A. mellifera*, other honeybees, bumblebees, and solitary bees), but also social wasps, with prevalence ranging from 2.2% to 67% prevalence [39]. Conversely, the ABPV host range is very limited. To date, ABPV has only been described in *A. mellifera*, *A. ceranae* [87], and bumblebees [88]. Our data show that *Vespidae* may be another host of such viruses. As a follow up, experimental infections will be required to assess the pathogenicity of these honey bee viruses (BQCV and ABPV) in hornets. 

If we assume that circulation of viruses in insect body should be related to the infection cycle, then *Vespa velutina* associated acypi-like virus sequences, identified in both brain and muscle from 140C and 140M and whose whole genome could be reconstructed, may be an infective virus. In aphids, APV is known to reduce growth, to increase developmental time and possibly related to an increased mortality rate [89]. Further experiments are needed to characterize its virulence in *Vespidae* and to bees, such as *A. mellifera*. 

Additionally, even if the whole *Vespa velutina* associated triato-like virus genome could not be reconstructed, the identification of sequences of this virus both in muscles and guts suggests that it may infect *V. velutina*. However, larger samplings are required to confirm its prevalence. Furthermore, the phylogenetic tree showed that these sequences are more similar to the *Bat dicistrovirus*, which has, to date, only been described twice in the feces of the bat species *Pipistrellus pipistrellus* [90,91]. Of note, this bat species has an insectivorous diet, which can explain the presence of this potentially insect-specific virus in their feces.

In contrast, the presence of viruses exclusively in the hornet gut may result from food contamination (infected preys or contaminated pollen) [38,53]. The absence of feces in cages where hornets were reared before freezing suggests that they could not eliminate the last food they ingested before sampling and sugar feeding. This could potentially lead to the detection of infected preys in the hornet gut content. Since DWV- C was only detected in the gut, we cannot state if they infect hornets or only their preys. The same conclusion can be drawn for all the other viruses described in this analysis, like Partiti-like virus 1, the *Motts Mill virus*, *Drosophila A virus* (DAV-like), the *Aphid lethal paralysis virus* (ALPV), the Bee Macula-like virus (BeeMLV), and Moku virus. The presence of Moku virus has already been described in an Asian yellow-legged hornet (*Vespa velutina nigrithorax*) collected in Belgium [33], but the viral metagenomics analysis was performed from a pool of 10 hornets, from which food contamination or real hornet infection could not be discriminated.

Our results from the French samples showed the presence of a large diversity of viruses in the invasive hornet *Vespa velutina*. DWV-B was predominant and may cause symptoms. Because DWV variants exhibited various pathogenicity levels, it is of great importance to assess their virulence for this new host species and to study their evolution over time, as recombination was already shown among variants [77]. Other viruses initially discovered in bees could infect the Asian hornet, since they were detected in different parts of the hornet body. The transmission routes from or to *V. velutina* have to be investigated to better assess the impact they may have on European honeybee and hornet populations. Moreover, new viruses were described, and the *Vespa velutina* associated acypi-like and *Vespa velutina* associated triato-like viruses were, for the first time identified in *Vespidae* species. In sum, there is an urgent need to study their host range and evaluate whether they represent a new threat to honeybees or a potential solution for the biocontrol of *V. velutina* in Europe, where they are dramatically spreading.

## Figures and Tables

**Table 1:** Sequencing and alignment statistics. RNA was extracted from brain (140C), muscle (159M (symptomatic hornet), 140M), or gut (144I).

Sample	Number of Read Pairs	Number of Contigs	Total Size of Contigs (in Nucleotides)	Read Alignment Rate (*)	Pair Alignment Rate (*)
159M	41,650,349	85,968	44,138,196	99.35%	71.34%
144I	50,537,821	468,927	276,819,205	98.44%	41.19%
140M	49,181,345	420,974	246,505,091	98.81%	51.54%
140C	49,526,919	820,766	374,354,293	99.08%	62.55%

(*) from samtools flagstat.

**Table 2:** Taxonomy and genomic characteristics of viruses detected in hornet samples.

Library	Virus Acronym	Virus Name	Family	Genus	Genome	Number of. Contig (s) Assembled	Contig(s) size	Expected Full-Length Genome Size (kb)	% Missing Genome	Coverage of Assembled Contigs *	Coverage of a Full-Length Expected Genome
159M	DWV-B	Deformed wing virus type B	Iflaviridae	Iflavirus	Nearly full-length	1	9434	10.1	6.7	11.1	10.4
140C	na	Vespa velutina associated acypi-like virus	unclassified	unclassified	Full-length	1	9872	10	0	535.1	535.1
	DWV-B	Deformed wing virus type B	Iflaviridae	Iflavirus	Nearly full-length	4	9665	10.1	4.1	8.5	8.2
	KBV	Kashmir bee virus	Dicistroviridae	Aparavirus	Partial	16	3536	9.5	62.9	1.9	0.7
140M	na	Vespa velutina associated acypi-like virus	unclassified	unclassified	Full-length	1	9962	10	0	1186	1186
	DWV-B	Deformed wing virus type B	Iflaviridae	Iflavirus	Nearly full-length	2	10000	10.1	0.8	13.2	13.2
	na	Vespa velutina associated triato-like virus	Dicistroviridae	Triatovirus	Partial	4	2380	9.3	74.3	4.5	1.2
144I	DWV-B	Deformed wing virus type B	Iflaviridae	Iflavirus	Full-length	1	10113	10.1	0	110,316.3	110,316.3
	na	Vespa velutina associated acypi-like virus	unclassified	unclassified	Full-length	1	9748	10	0	122	122
	ABPV	Acute bee paralysis virus	Dicistroviridae	Aparavirus	Full-length	1	9474	9.5	0	1794.6	1794.6
	BQCV	Black queen cell virus	Dicistroviridae	Triatovirus	Full-length	1	8464	8.6	0	268.4	268.4
	na	Vespa velutina associated Menton virus	unclassified	unclassified	Full-length segment 1	1	3031	2.8 (seg.1)	0 (Seg 2 missing: 1.5kb)	316.7	316.7
	na	Vespa velutina associated ifla-like virus	unclassified	unclassified	Full-length	1	10023	9.9	0	19.5	19.5
	na	Vespa velutina associated permutotetra-like virus 1	unclassified	unclassified	Full-length	1	4851	4.8	0	34.7	34.7
	na	Vespa velutina associated permutotetra-like virus 2	unclassified	unclassified	Full-length	1	4742	4.8	0	11.7	11.7
	na	Vespa velutina associated partiti-like virus 1	Partitiviridae	unclassified	Full-length segment 1	1	1779	1.7	0 (Seg 2 missing: 1.7 kb)	1082.5	1082.5
	na	Vespa velutina associated partiti-like virus 2	Partitiviridae	unclassified	Full-length segment 1	1	1415	1.4	0 (Seg 2 missing: 1.7 kb)	44.6	44.6
	na	Vespa velutina associated partiti-like virus 3	Partitiviridae	unclassified	Full-length segment 1	1	1417	1.4	0 (Seg 2 missing: 1.7 kb)	100.3	100.3
	na	Vespa velutina associated partiti-like virus 4	Partitiviridae	unclassified	Partial segment 1	1	467	1.5	Seg1: 63.2 (Seg 2 missing: 1.7 kb)	6.2	1.9
	na	Vespa velutina associated nora-like virus	unclassified	unclassified	Partial	1	1406	8	17.5	6.6	1.2
	na	Vespa velutina associated triato-like virus	Dicistroviridae	Triatovirus	Partial	3	3149	9.3	66	4.9	1.6
	ALPV	Aphid lethal paralysis virus	Dicistroviridae	Cripavirus	Partial	20	4186	9.8	57.4	2	0.9
	na	Moku virus	Iflaviridae	Iflavirus	Partial	23	6232	10.1	38	2	1.3
	BMLV	Bee Macula-like virus	Tymoviridae	na	Partial	13	3102	6.3	50.3	2.4	1.2
	DWV-C	Deformed wing virus type C	Iflaviridae	Iflavirus	Partial	15	3292	10.1	64.9	9,634.2	3,377

* Coverage calculated from the corresponding full-length expected.

## Supplementary Materials

The following are available online at https://www.mdpi.com/1999-4915/11/11/1041/s1. Table S1: Hornet and honey bee sample list and corresponding viral PCR detections. Table S2: Accessions number and virus names of sequences used in virus phylogenies. Table S3: Polymorphism and expression levels of viral sequences between samples. Table S4: Read counts (and read %) supporting SNP from Vespa velutina-associated acypi-like virus across hornet samples. Figure S1: Kraken counts from reduced fasta contigs for brain (140C), muscle (140M, 159M) or gut (144I) samples. Only the most significant values and honey bee-associated viruses are shown. Figure S2: Quality plots of RNAseq reads of *Vespa velutina* samples 140C and 140M before FastQC trimming. Figure S3: Quality plots of RNAseq reads of *Vespa velutina* samples 144I and 159M before FastQC trimming. Figure S4: Schematic representation, coverage and annotation of complete *Deformed wing virus* genome found in hornets. (a–d): *Deformed wing virus* strain B found in 140C, 140M, 144I, and 159M, respectively. Figure S5: Multiplex PCR virus detection for six hornet and four bee samples originating from each apiary (Table S1). Ladder 100 bp is from Promega, the most intense band corresponds to a 500-bp sized fragment. Figure S6: Schematic representation, coverage and annotation of complete or partial genomes of RNA honey bee-associated viruses found in hornets. (a) *Aphid lethal paralysis virus*-like, (b) Bee macula-like virus, (c) Moku virus, (d) *Kashmir bee virus*, (e) *Acute bee paralysis virus* and (f) *Black queen cell virus*. Figure S7: Maximum likelihood amino acid phylogeny of Bee macula-like virus replicase from hornets (243 aa, LG+I+G+F model). GenBank accessions are shown in Table S2. Figure S8: Maximum likelihood amino acid replicase phylogeny of (a) *Acute bee paralysis virus* (139 amino acids, JTT+G model) and (b) *Black queen cell virus* (8,683 aa, GTR+G+I model). GenBank accessions are shown in Table S2.
