# Supplementary material for: Viruses in the Invasive Hornet Vespa velutina
Source: Viruses. 2019 Nov 8;11(11):1041. doi: 10.3390/v11111041 (PMC6893812; doi:10.3390/v11111041)
Supplement: Supplementary file 1 [file viruses-11-01041-s001.zip › Figure S1.pptx]

## Slide 1
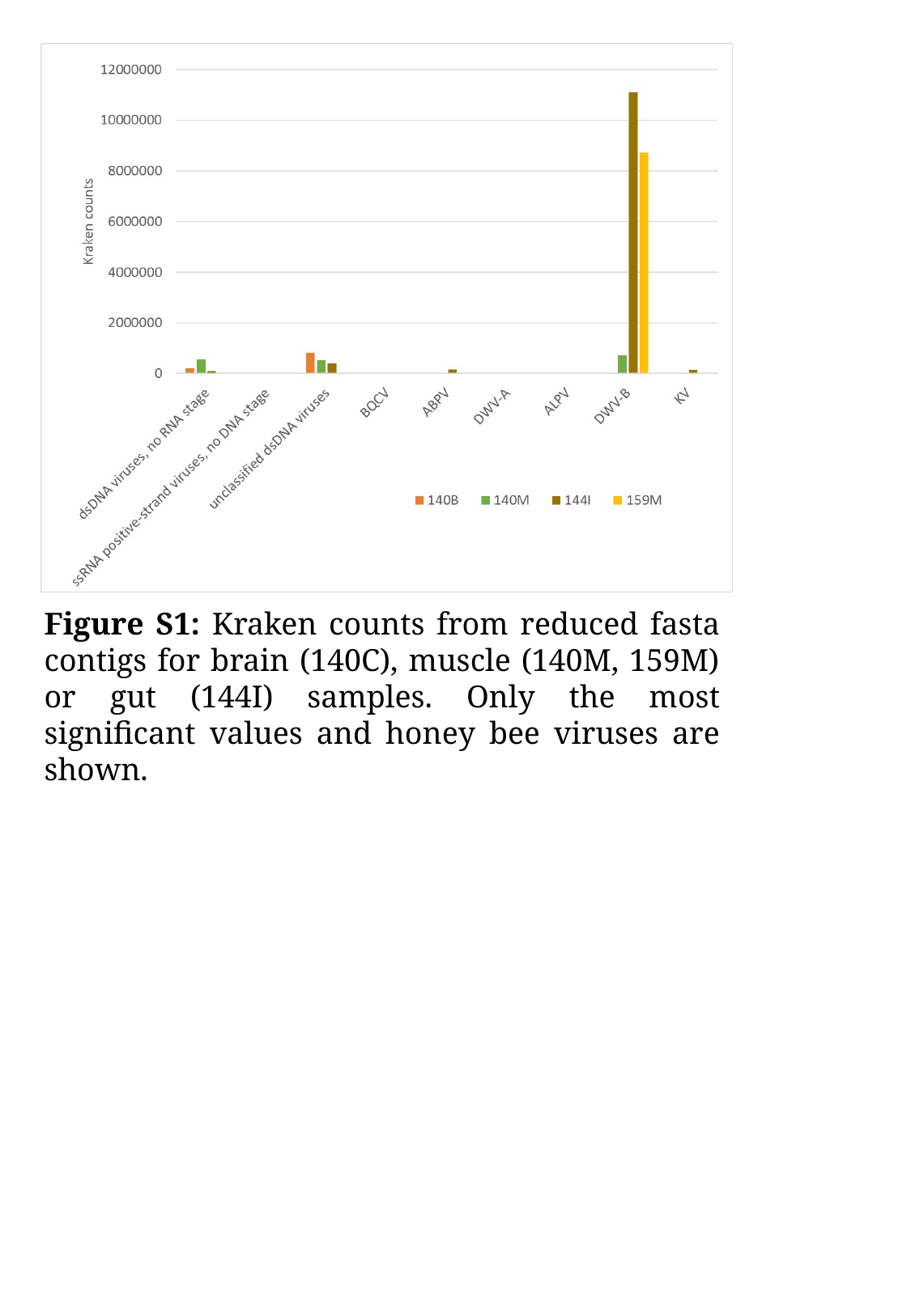

Figure S1: Kraken counts from reduced fasta contigs for brain (140C), muscle (140M, 159M) or gut (144I) samples. Only the most significant values and honey bee viruses are shown.
