# Supplementary material for: Viruses in the Invasive Hornet Vespa velutina
Source: Viruses. 2019 Nov 8;11(11):1041. doi: 10.3390/v11111041 (PMC6893812; doi:10.3390/v11111041)
Supplement: Supplementary file 1 [file viruses-11-01041-s001.zip › Figure S2.pptx]

## Slide 1
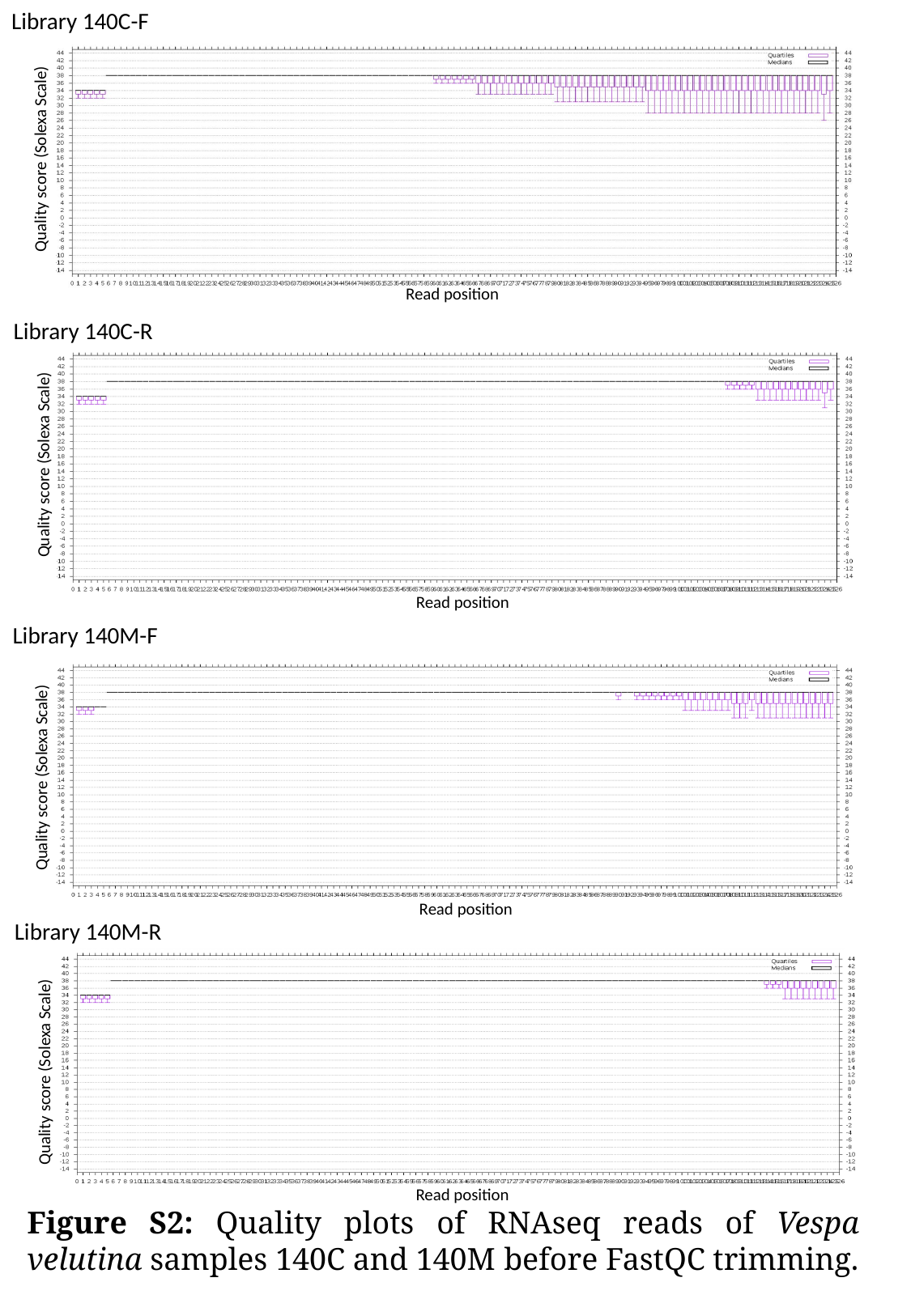

Library 140C-F
Quality score (Solexa Scale)
Read position
Library 140C-R
Quality score (Solexa Scale)
Read position
Library 140M-F
Quality score (Solexa Scale)
Read position
Library 140M-R
Quality score (Solexa Scale)
Read position
Figure S2: Quality plots of RNAseq reads of Vespa velutina samples 140C and 140M before FastQC trimming.
