# Supplementary material for: Viruses in the Invasive Hornet Vespa velutina
Source: Viruses. 2019 Nov 8;11(11):1041. doi: 10.3390/v11111041 (PMC6893812; doi:10.3390/v11111041)
Supplement: Supplementary file 1 [file viruses-11-01041-s001.zip › Figure S3.pptx]

## Slide 1
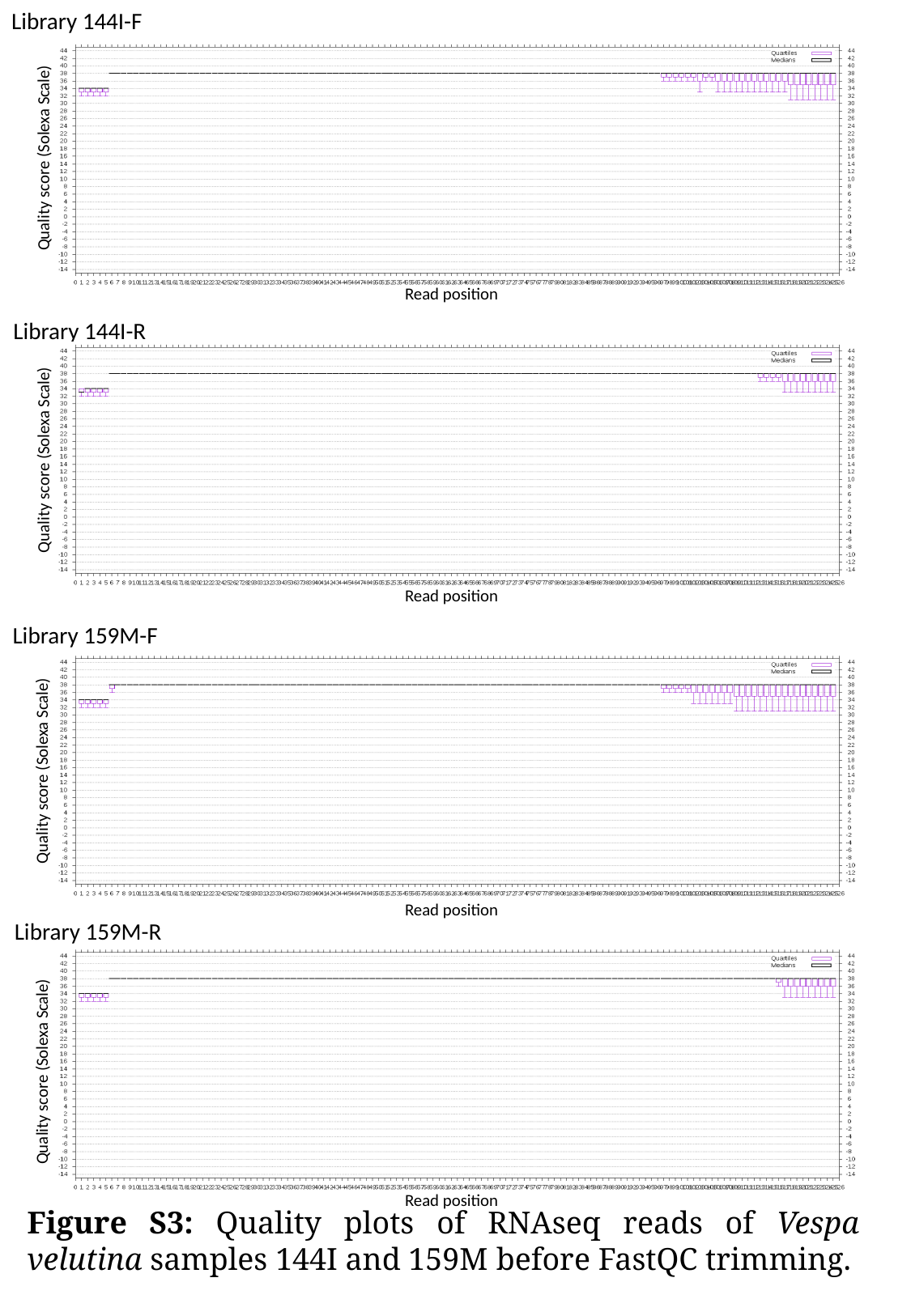

Library 144I-F
Quality score (Solexa Scale)
Read position
Library 144I-R
Quality score (Solexa Scale)
Read position
Library 159M-F
Quality score (Solexa Scale)
Read position
Library 159M-R
Quality score (Solexa Scale)
Read position
Figure S3: Quality plots of RNAseq reads of Vespa velutina samples 144I and 159M before FastQC trimming.
