# Supplementary material for: Viruses in the Invasive Hornet Vespa velutina
Source: Viruses. 2019 Nov 8;11(11):1041. doi: 10.3390/v11111041 (PMC6893812; doi:10.3390/v11111041)
Supplement: Supplementary file 1 [file viruses-11-01041-s001.zip › Figure S4.pptx]

## Slide 1
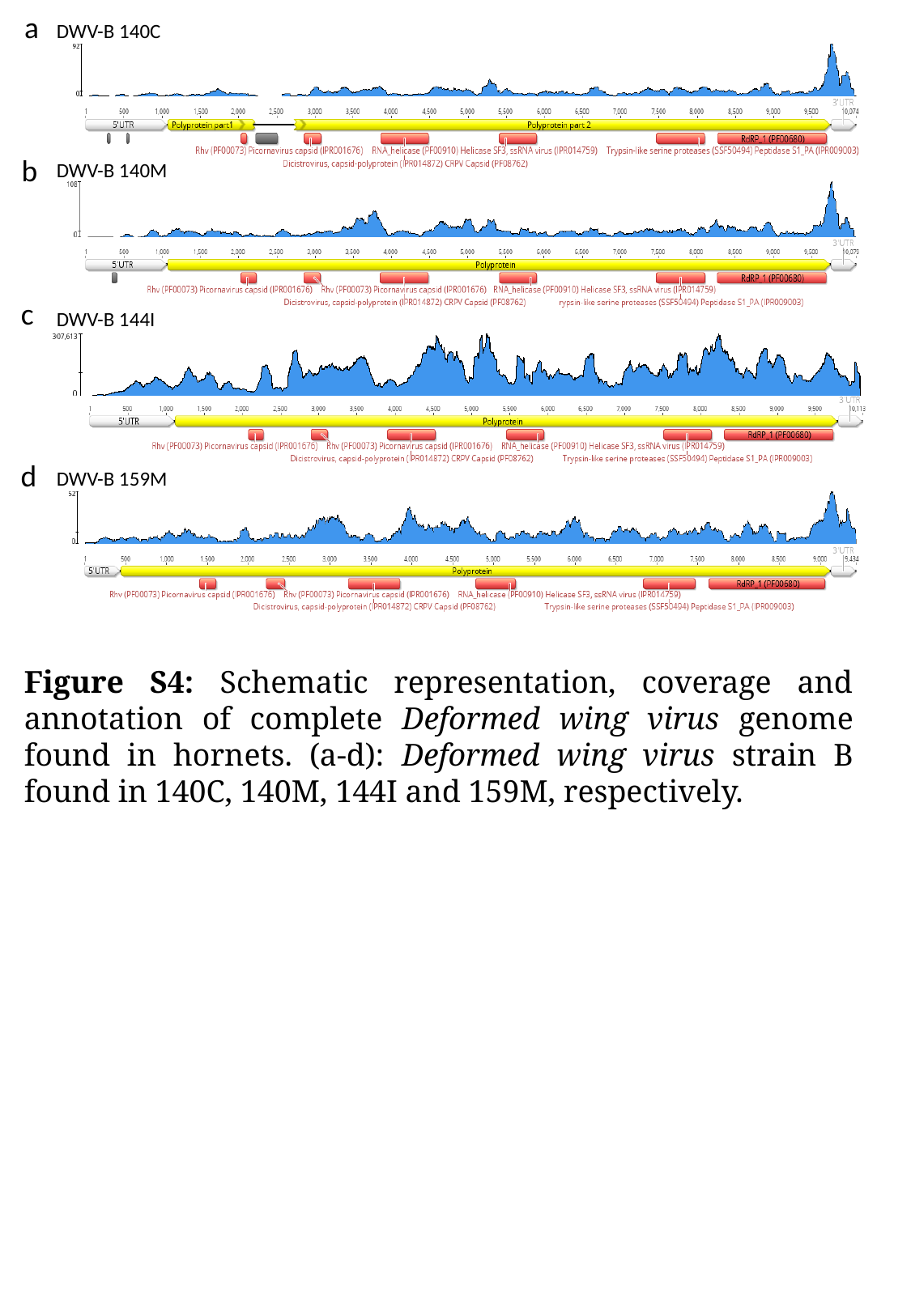

a
DWV-B 140C
b
DWV-B 140M
c
DWV-B 144I
d
DWV-B 159M
Figure S4: Schematic representation, coverage and annotation of complete Deformed wing virus genome found in hornets. (a-d): Deformed wing virus strain B found in 140C, 140M, 144I and 159M, respectively.
