# Supplementary material for: Viruses in the Invasive Hornet Vespa velutina
Source: Viruses. 2019 Nov 8;11(11):1041. doi: 10.3390/v11111041 (PMC6893812; doi:10.3390/v11111041)
Supplement: Supplementary file 1 [file viruses-11-01041-s001.zip › Figure S5.pptx]

## Slide 1
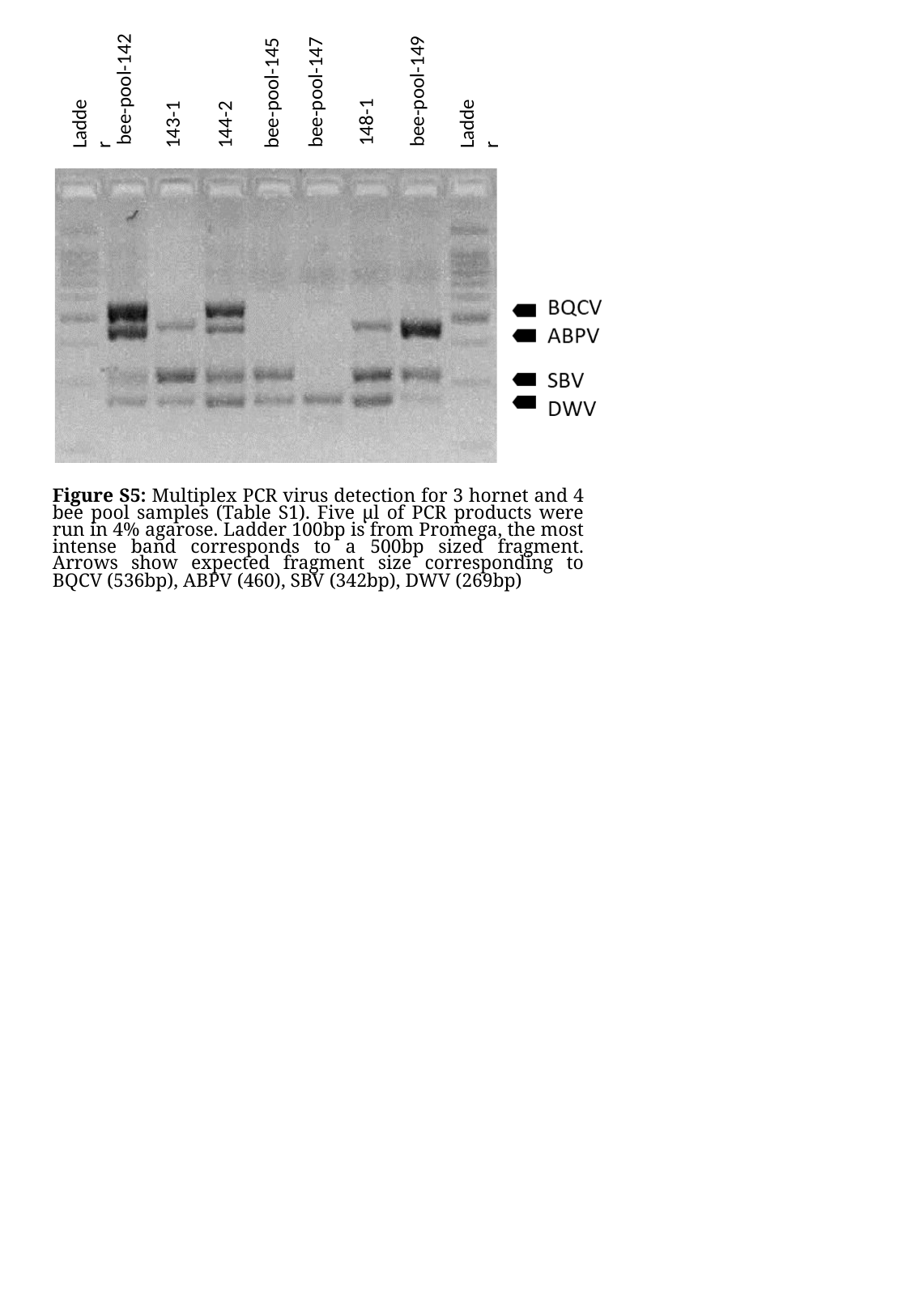

bee-pool-149
bee-pool-147
bee-pool-142
bee-pool-145
144-2
148-1
143-1
Ladder
Ladder
Figure S5: Multiplex PCR virus detection for 3 hornet and 4 bee pool samples (Table S1). Five µl of PCR products were run in 4% agarose. Ladder 100bp is from Promega, the most intense band corresponds to a 500bp sized fragment. Arrows show expected fragment size corresponding to BQCV (536bp), ABPV (460), SBV (342bp), DWV (269bp)
