# Supplementary material for: Viruses in the Invasive Hornet Vespa velutina
Source: Viruses. 2019 Nov 8;11(11):1041. doi: 10.3390/v11111041 (PMC6893812; doi:10.3390/v11111041)
Supplement: Supplementary file 1 [file viruses-11-01041-s001.zip › Figure S6.pptx]

## Slide 1
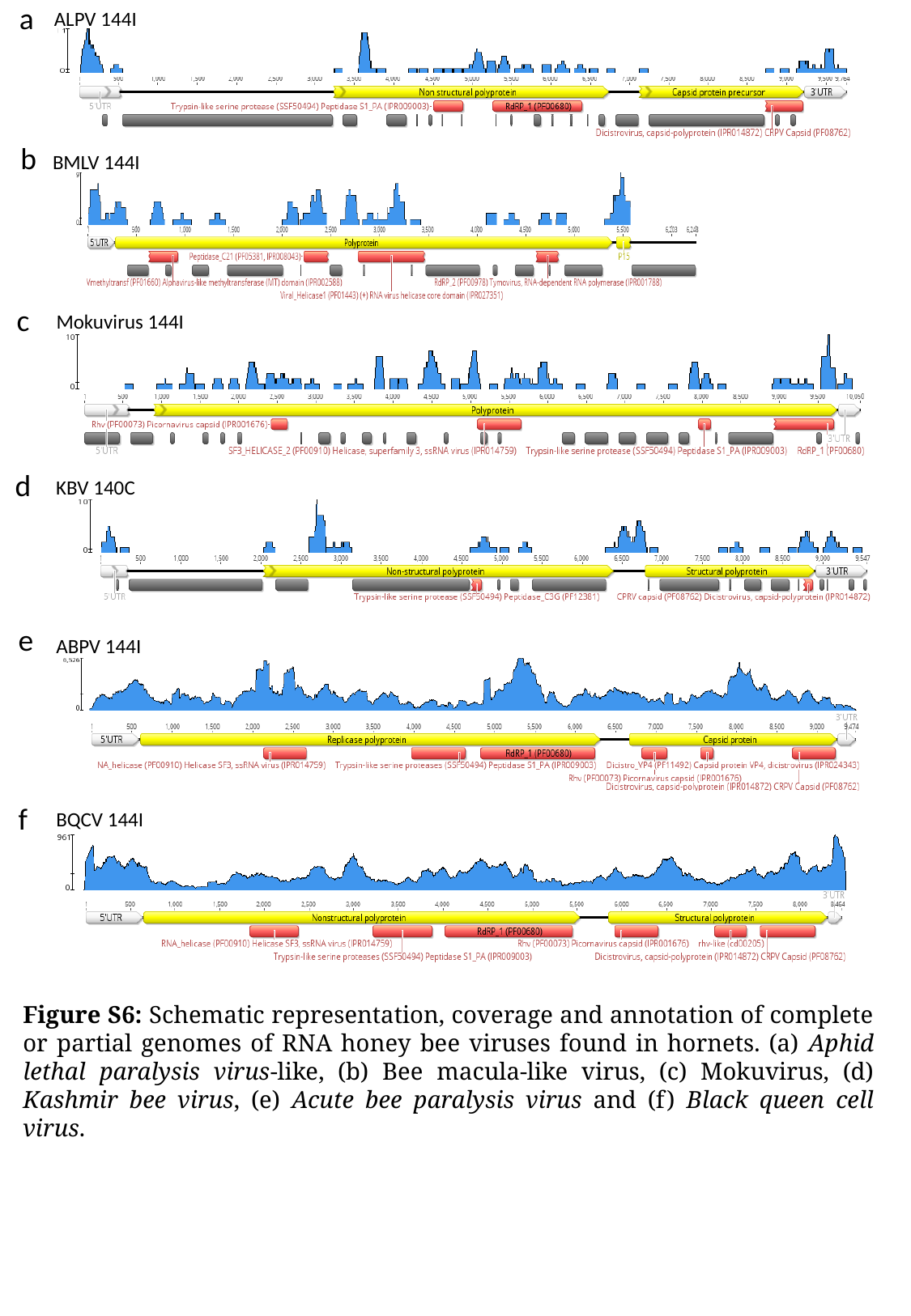

a
ALPV 144I
b
BMLV 144I
c
Mokuvirus 144I
d
KBV 140C
e
ABPV 144I
f
BQCV 144I
Figure S6: Schematic representation, coverage and annotation of complete or partial genomes of RNA honey bee viruses found in hornets. (a) Aphid lethal paralysis virus-like, (b) Bee macula-like virus, (c) Mokuvirus, (d) Kashmir bee virus, (e) Acute bee paralysis virus and (f) Black queen cell virus.
