# Supplementary material for: Viruses in the Invasive Hornet Vespa velutina
Source: Viruses. 2019 Nov 8;11(11):1041. doi: 10.3390/v11111041 (PMC6893812; doi:10.3390/v11111041)
Supplement: Supplementary file 1 [file viruses-11-01041-s001.zip › Figure S7.pptx]

## Slide 1
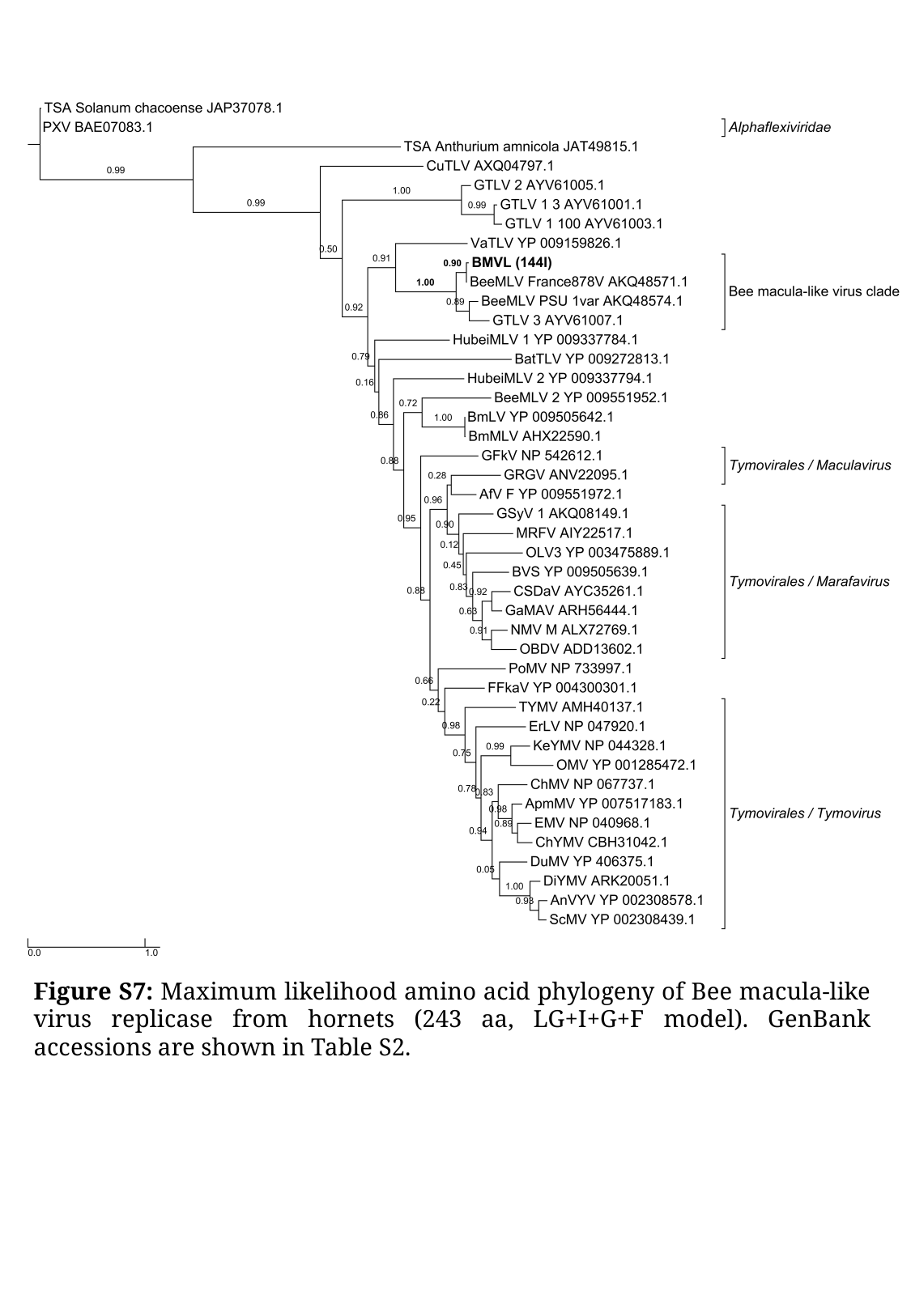

Figure S7: Maximum likelihood amino acid phylogeny of Bee macula-like virus replicase from hornets (243 aa, LG+I+G+F model). GenBank accessions are shown in Table S2.
