# Supplementary material for: Viruses in the Invasive Hornet Vespa velutina
Source: Viruses. 2019 Nov 8;11(11):1041. doi: 10.3390/v11111041 (PMC6893812; doi:10.3390/v11111041)
Supplement: Supplementary file 1 [file viruses-11-01041-s001.zip › Figure S8.pptx]

## Slide 1
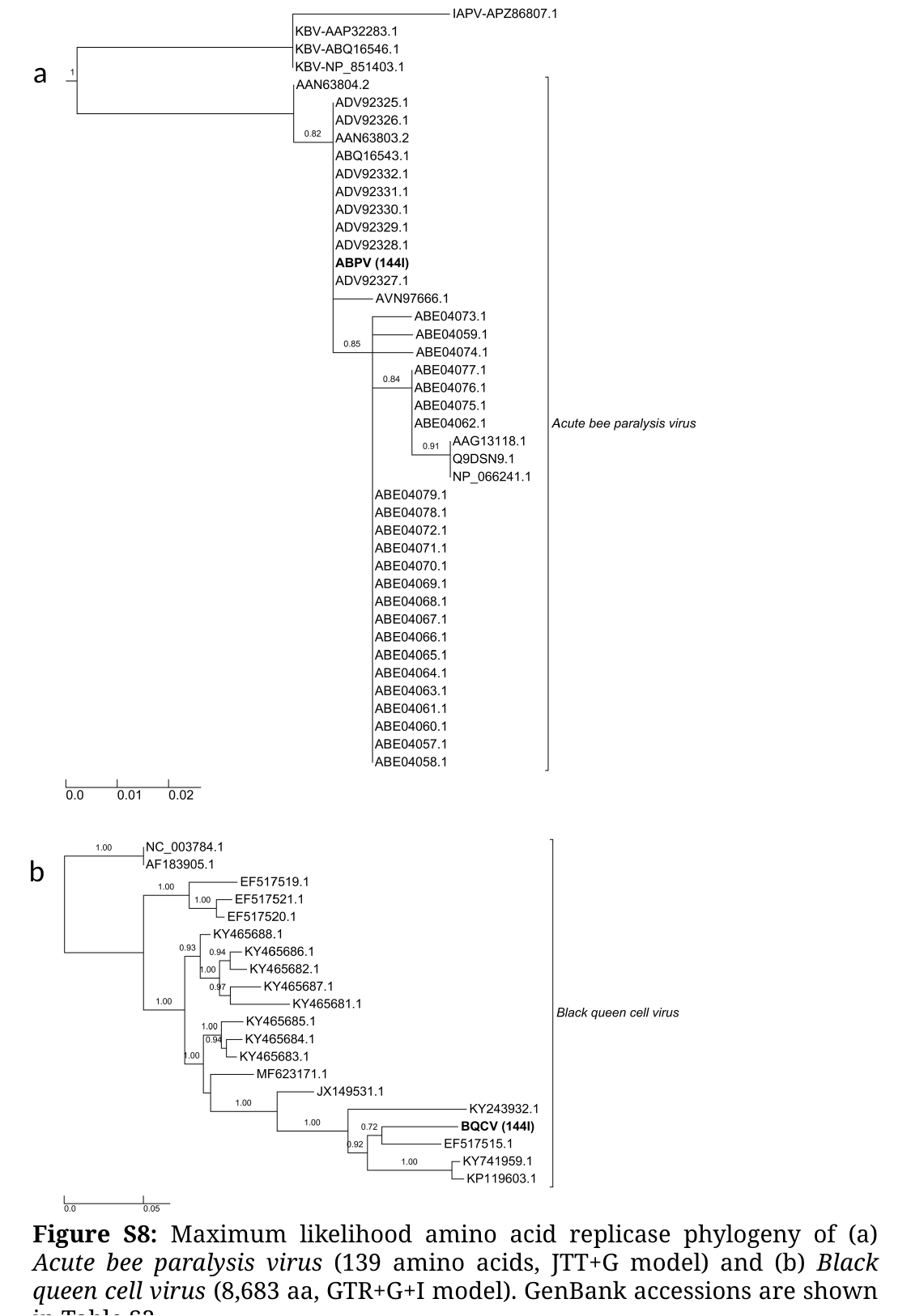

a
b
Figure S8: Maximum likelihood amino acid replicase phylogeny of (a) Acute bee paralysis virus (139 amino acids, JTT+G model) and (b) Black queen cell virus (8,683 aa, GTR+G+I model). GenBank accessions are shown in Table S2.
